# An Exploratory Study of Verbal and Non-Verbal Communication in Saudi Arabian Families

**DOI:** 10.3390/bs13020175

**Published:** 2023-02-15

**Authors:** Lowai G. Abed, Mohaned G. Abed, Todd K. Shackelford

**Affiliations:** 1 Department of Communication and Public Relations, College of Communication and Media, University of Jeddah, Jeddah 23218, Saudi Arabia; 2 Department of Special Education, Faculty of Educational Graduate Studies, King Abdulaziz University, Jeddah 21589, Saudi Arabia; 3 Department of Psychology, Oakland University, Rochester, MI 48309, USA

**Keywords:** familial relations, verbal communication, non-verbal communication, gender, age, monthly income, level of education, Saudi Arabia

## Abstract

This exploratory study investigated whether factors such as gender, age, level of education, monthly income, and the number of family members are associated with verbal and non-verbal communication in Saudi Arabian families. A convenience sampling procedure was used to recruit 182 Saudi Arabian adults who responded to a self-report survey. Verbal and non-verbal communication was categorized into positive and negative communication. Descriptive and ordinal regression analyses were conducted to assess the relationships of familial variables with communication. Female gender status had a small negative association with positive communication, whereas the number of family members, level of education, monthly income, and age did not correlate with positive communication or negative communication. The Discussion section addresses the limitations of the current study and identifies several directions for future research, with special attention to the Saudi Arabian family context.

## 1. Introduction

### 1.1. Communication in Families

Familial factors can affect how people communicate with family members [1]. Guided by the results of previous research [2,3,4], the current study tested the hypothesis that familial factors such as socioeconomic status, level of education, gender, and age (of the parents), as well as the number of family members, are associated with how family members communicate with one another verbally and non-verbally [5]. For instance, highly educated parents with a high monthly income are more tolerant (i.e., authoritative) as opposed to controlling (i.e., authoritarian) in their communications with their children [6]. Communication styles, in turn, can affect the health and well-being of family members. One study found that family communication directly affected adolescents’ mental health through their body-image satisfaction and indirectly affected adolescents’ mental health through their self-esteem [7]. Previous research investigating the interrelationships of familial factors and communication has focused on Western cultures and families [6,8,9]. The current study extends this research to Middle Eastern samples, specifically Saudi Arabian families.

### 1.2. Verbal Communication

Verbal communication relies on the oral transmission of words to relay messages [10]. Relative to non-verbal communication, verbal communication is direct, leaving less opportunity for the misinterpretation of a message. Studies that investigate children’s mental health within a family often consider verbal communication. Aloia [2] studied the influence of family schemas, parental support, and verbal aggression on the mental health of adolescents. The researcher found that family conversation and parental support were positively correlated with adolescent mental health. On the other hand, adolescents in families that require conformity, such as those in families that engage in verbal aggression, reported lower mental health [2].

In the helping professions, components of communication are the basis for building a therapeutic alliance, a relational facet that envisages psychotherapy outcome and change as being shaped by patient–therapist interaction from the first stages of the encounter to the conclusion of psychotherapy [4]. One study on therapist–patient encounters documented that the therapist’s verbal communication (asking and searching) and non-verbal communication (listening and politely interrupting) and a depressed patient’s verbal communication (affirming, sharing, and exploring) and non-verbal communication (conveying emotion and politely interrupting) reflect patterns of involvement with mutual behavior linked to the reciprocation of each participant [4]. Rocco et al. (2003, cited in [4]) found dynamic verbal and non-verbal communication to be the basis of a positive therapeutic alliance that relies on mutual interaction between the contributions of the therapist and patient.

Some studies have explored how economic challenges and parental communication behaviors are associated with mental health issues among adolescents. Rodriguez et al. [11] investigated the associations between socioeconomic status, parental communication, and mental health among adolescents in an urban setting and found that positive parental communication (talking openly and frequently, providing clear information, listening, and being responsive) was linked with decreased adolescent risk-taking behavior (substance use, alcohol intake, and brief sexual relationships) and fewer mental health problems. Moreover, positive parental communication has been linked with positive parenting behavior such as warmth and nurturance, often studied in the context of the family stress model [11]. Thus, how a parent communicates with his or her children may be a protective factor in adolescent mental health.

In a study of the effects of family-oriented communication skills training on mental health problems such as depression and anxiety, Ghazavi et al. [3] found that communication skills among older individuals predicted their mental health. For instance, poor communication skills not only predicted anxiety and distress in older adults but also predisposed them to social isolation. Here, the sender or initiator of communication is likely to be isolated due, in part, to their inability to communicate well with others [3]. Similarly, if power dynamics are involved, as in a parent–child relationship, then the child is likely to be forced to listen to a parent with poor communication skills and this may affect the child’s mental health [3]. Thus, there may be mental health implications of poor communication skills within a family due to authority and power dynamics. There is a need to resolve familial conflicts to maintain family stability, and this is more likely with appropriate, effective, and skilled verbal communication [3].

### 1.3. Non-Verbal Communication

Non-verbal communication has a greater chance of being misinterpreted than verbal communication due to cultural and other contextual factors. However, there are non-verbal communication cues that are considered standard, normal reactions to specific stimuli—particularly facial expressions. Grebelsky-Litchman and Shenker [6] studied the patterns of non-verbal communication in social and situational contexts and found that non-verbal communication affects parent–child interactions. Some of the situational and contextual factors investigated by Grebelsky-Litchman and Shenker that may affect non-verbal parental communication included socializing factors, such as a family’s socioeconomic status (SES) and gender (of the parents and children). With regard to SES, the study found that SES is associated with specific patterns of parental control [6]. Bloomguist (2009, cited in [6]) reported that high-SES parents were more verbally communicative with their children than were low-SES parents. In addition, high-SES (compared with low-SES) parents used both verbal and non-verbal communication in a manner that facilitates child development [6].

With regard to gender, a parent’s gender contributes to how the parent and child interact. For instance, mothers are more sensitive to non-verbal communication from their children, which they interpret more accurately than do fathers [6]. Fathers, on the other hand, expressed more definitive non-verbal cues than did mothers. Additionally, fathers displayed a greater propensity than mothers to adopt authoritarian parenting [6]. Parental communicative behaviors have also been studied with regard to the child’s gender. Lytton and Romney (2010, cited in [6]) noted that the presumption that boys have more disciplinary issues than girls influences parental behavior to be more strict, punitive, and severe towards sons than daughters. Differential treatment in terms of parental non-verbal communication toward daughters and sons might be attributed, in part, to gender stereotypes held by parents (Lytton and Romney, 2010, cited in [6]). As such, girls are likely to receive more support, encouragement, and physical contact than boys within a family (Lytton and Romney, 2010, cited in [6]).

Spapé et al.’s [12] investigation of the semiotics of the message and messenger indicated that non-verbal communication affects how people interpret overt, verbal messages. For instance, facial expressions and social touch encourage affinity, empathy, and agreement. Determining what a message means (i.e., semiotics) relies on different modalities and contextual features. For instance, language can influence emotion perception by activating brain areas that facilitate interpreting a message as either literal or ironic [12].

Guided by the results of previous research, the current study investigated whether familial factors such as gender and age (of parent and child) affect verbal and non-verbal communication within the context of Saudi Arabian families. We secured self-report survey data from a sample of Saudi Arabian adults. We categorized verbal and non-verbal communications into positive communication and negative communication with members of the family, with a special focus on parent–child communication. This exploratory study thus extends previous research with Western samples on familial factors associated with verbal and non-verbal communication, to the Saudi Arabian family context.

## 2. Methods

### 2.1. Participants

A total of 182 Saudi Arabian adults identified by convenience sampling completed a self-report survey. We discarded incomplete surveys from 19 prospective participants. Table 1 displays a summary of the available demographic attributes for the sample. The participants were members of the general public and had varied demographic attributes along age, gender, level of education, and monthly income. More females than males participated. In addition, the age group between 41 and 50 years included the greatest number of participants, and the most frequent educational level of participants was the completion of a bachelor’s degree. All participants reported that they had at least 5 family members, and their response in this case was coded “1”.

### 2.2. Procedure

The sampling procedure was pseudo-random and guided by convenience, but it was also purposive in that it did not include children as participants. The pseudo-random quality of the sampling was facilitated by the broad demographic targeting reflected in the survey, such that both genders were targeted, alongside respondents of different educational backgrounds, monthly income, etc. The survey was constructed in Google Forms and a recruitment announcement was distributed online via the social media platforms WhatsApp, Twitter, and Snapchat. Prospective participants who met participation requirements were provided with a link to the online survey. All responses were provided confidentially and without reward.

### 2.3. Materials

In the section addressing communication, participants completed 16 items addressing communication on a Likert-type scale anchored by 1 = “strongly disagree” and 5 = “strongly agree.” These items were drawn from the McMaster Family Assessment Device, a validated measure of familial interaction and communication [13,14]. Participants were instructed: “Please read each statement carefully, and decide how well it describes your own family.” These items were categorized into positive communication (PC) and negative communication (NC), with each category containing items with elements (verbal and non-verbal) that previous research (reviewed above) suggested may be associated with family functioning. Table 2 displays the communication items and their associated study codes.

## 3. Results

We first present the descriptive demographic statistics for the sample, followed by descriptive statistics for the items assessing communication. Participant demographic attributes including age, gender, and monthly income were coded to facilitate inclusion in regression analysis as shown in Table 3. The descriptive statistics focused on the mean and the standard deviation as shown in Table 4. Table 5 presents descriptive statistics for the communication items. Negative communication items NC1 (“Planning family activities is difficult because we misunderstand each other.”) and NC5 (“You only get the interest of others when something is important to them.”) had the highest response score for the communication items.

### Ordinal Regression Results

Ordinal regression analyses were conducted to predict mean composite scores for positive communication and negative communication (Cronbach’s *α*s > 0.80), with age, gender, level of education, monthly income, and number of family members included as predictors. Table 6 and Table 7 presents the key results for positive and negative communication, respectively. A reviewer suggested we use ranks for income. The results of these analyses (available on request) did not differ substantively from the results presented in Table 6 and Table 7.

The relationships were weak both for positive communication and negative communication. Level of education, monthly income, and age have no relationship with positive communication based on the coefficient results. On the other hand, gender had a weak negative relationship with positive communication, and the number of family members had no relationship with the same. For negative communication, as gender value increased, positive communication decreased. The coded value of gender was “0” and “1” as shown in Table 3. This can be interpreted to mean that women produce less positive communication than did men.

The number of family members had a weak but non statistically significant positive relationship with negative communication, whereas monthly income, age, and gender did not display relationships with negative communication. Additionally, level of education had no relationship with negative communication.

## 4. Discussion

A key hypothesis explored in this study is that, in the Saudi Arabian context, familial factors such as socioeconomic status, level of education, gender, and the number of family members affect how members of a household (parents and children) communicate verbally and non-verbally. For instance, highly educated Western parents with a high monthly income are more tolerant (i.e., authoritative) as opposed to controlling (i.e., authoritarian) in their communications with their children [6]. The current results suggest that female gender status is associated with negative communication. However, in the current Saudi Arabian familial context, no other demographic variables are associated with positive communication or negative communication.

Several factors not investigated in the current study, such as parent’s personality traits and child’s personality traits, might be explored in future research, as they might affect communication patterns in Saudi Arabian families [3,4,15]. The majority of previous work in this area has focused on Western samples. The current study investigated communication in Saudi Arabian households, and future research might explore these relationships in other cultural contexts, including in Asian and African samples. We note as a limitation of the current research that our sample size was not large. However, we suggest that it was sufficient to conduct the analyses we report. With this in mind, we nevertheless suggest that future research secures data from a larger sample, to ensure robust results.

Gender is, in part, a social and cultural construction and can affect how people communicate with one another, not just in the family but also in the community [16,17]. One of the findings of the current study is that female gender status is associated negatively with positive communication, and this may have implications for the mental health of family members. If a parent communicates negatively, then this may affect others, especially children and adolescents [2]. Because of the small effect size and because we did not have a strong theoretical rationale for predicting that female gender status would negatively associate with positive communication, we suggest that research first attempts to replicate this finding in an independent Saudi Arabian sample of adults.

Few studies have investigated the impact of the number of family members on communication patterns, and no previous research has investigated this in the context of Saudi Arabian families. We did not find statistically significant relationships between the number of family members and either positive communication or negative communication in Saudi Arabian families. The presence of a greater number of family members is nevertheless intriguing and warrants exploration in future research with larger sample sizes because of the potential impact and interaction among different personalities in the family. This influence of personality on communication can be addressed by social psychological and humanistic theories. Social psychological theory posits that human behavior is a result of the interaction of mental states and social situations [18]. Therefore, as more people in a household interact, the chances of being affected increase, although the outcomes of such interactions may depend on a person’s attributes. Rogers’ humanistic theory argues that for individuals to grow psychologically, they need a setting that provides them with authenticity (i.e., honesty and self-disclosure), acceptance, and understanding [19]. Both verbal and non-verbal communication presented by different personalities in a household may affect the communication patterns of family members, and these communication patterns may have downstream consequences for mental health. Interestingly, Grinde and Tambs [20] conducted a study of Norwegian households in which they found that a larger household (i.e., greater number of family members) is associated with fewer mental health problems in children. Moreover, the closer the siblings were in age, the more pronounced the positive effects [20].

Ennis and Bunting [5] investigated personal and socio-demographic factors, such as age, education, and household income, and found that these factors predicted communication patterns and susceptibility to mental health problems. Moreover, larger families with illiterate parents and lower income were more vulnerable to psychological issues arising from family financial burdens [5]. In the current study, we assessed how educated versus uneducated parents communicate with their children. Parents with more education were more likely to communicate positively, although this result was not statistically significant. Respondents with a diploma or a bachelor’s degree comprised the group with the highest qualifications. McFarland and Wagner’s [21] study addressing college education and depressive symptoms among American young adults found a negative association between completing a college degree and depressive symptoms, although the study did not consider the role of personality traits or other individual difference variables. In other words, individuals with a college degree had lower levels of depressive symptoms compared with individuals with a high school degree or lower.

Cultural factors can influence how parents and children communicate in different countries. Fakhrunnisak and Patria’s [9] longitudinal study of the effects of parents’ education on children’s happiness in Indonesia found that although fathers’ education influenced happiness in children and spouses, there was no significant relationship between mothers’ education and children’s happiness. This suggests that although parents’ education levels were associated with their children’s mental health, there are different relationships found through the different groupings of parent and child gender [9].

Although the current study found that age is not associated with positive communication, the study did not consider the role of older family members (i.e., those older than 60 years). This group is composed of grandparents or other elderly relatives. One study investigating the effect of family-oriented communication skills training on mental health in older individuals found that such skills can reduce depression, anxiety, and stress in the elderly [3]. This highlights the significance of family-oriented communication skills in alleviating stress that may cause mental health problems. A study similar to the current study investigated associations between family communication patterns and mental health and found that a “conversation” dimension predicted children’s mental health whereas a “conformity” dimension did not predict children’s mental health [16]. This showcases that the type of communication can affect the recipient.

## 5. Conclusions

Verbal and non-verbal patterns of communication (both positive and negative) were hypothesized to be affected by several familial factors, namely age, gender, level of education, monthly income, and the number of family members. However, the results show that there is no relationship between either type of communication and age, level of education, and monthly income. Gender displayed a small negative relationship with positive communication. Cultural norms and expectations may affect how parents of each gender communicate with their children. In the case of Saudi Arabia, a cultural shift from traditional Islamic culture to acclimatization to Western culture has been viewed as a possible cause of cultural conflict for women, in particular. Moreover, for women more than men, widowhood sometimes causes financial challenges that can produce mental health problems, as can domestic violence victimization. Both phenomena may place greater mental health burdens on women than men, and these mental health issues can negatively impact communication with children and other family members. The measure of the number of family members is relatively new and the few studies that have investigated this indicate that children from larger families experience more positive communication and better mental health, perhaps due to the presence of older siblings. Future research might include a comparative study of family communication patterns with a focus on the cultural norms of different countries to determine the role of culture factors on the communication patterns and mental health of families.

## Figures and Tables

**Table 1:** Summary of the available demographic attributes of the sample.

Variable	Attributable	Total
Age	Less than 20	11
	21–30	17
	31–40	44
	41–50	63
	51–60	39
	More than 60	8
	**N**	**182**
Gender	Male	71
	Female	111
	**N**	**182**
Level of Education	Elementary school	7
	Middle school	19
	High school	21
	Diploma	48
	Bachelor’s degree	61
	Master’s degree	19
	Doctoral degree	7
	**N**	**182**
Monthly income	No income/don’ know	14
	Less than 5000	39
	5000–9999	47
	10,000–14,999	54
	15,000–20,000	22
	More than 20,000	6
	**N**	**182**
Number of family members	Less than 5	84
	More than 5	98
	**N**	**182**

Note: Currency is SAR.

**Table 2:** Communication items and study codes.

Code	Items
PC1	In times of crisis we can turn to each other for support.
PC2	We cry openly.
PC3	We make sure members meet their family responsibilities.
PC4	We confide in each other.
PC5	Individuals are accepted for what they are.
PC6	People come right out and say things instead of hinting at them.
PC7	We resolve most of everyday problems around the house.
PC8	When someone is upset the other knows why.
	
NC1	Planning family activities is difficult because we misunderstand each other.
NC2	When you ask someone to do something, you have to check that they did it.
NC3	We are reluctant to show our affection for each other.
NC4	We cannot talk to each other about the sadness we feel.
NC5	You only get the interest of others when something is important to them.
NC6	You can’t tell how a person is feeling from what they are saying.
NC7	Some of just don’t respond emotionally.
NC8	We avoid discussing our fears and concerns.

**Table 3:** Coded independent variables to be used for regression analysis.

Variable	Attribute	Code
Age	Less than 20	0
	21–30	1
	31–40	2
	41–50	3
	51–60	4
	More than 60	5
Gender	Male	0
	Female	1
Level of education	Elementary school	0
	Middle school	1
	High school	2
	Diploma	3
	Bachelor’s degree	4
	Master’s degree	5
	Doctoral degree	6
Monthly income	Less than 5000	0
	5000–9999	1
	10,000–14,999	2
	15,000–20,000	3
	More than 20,000	4
	No income/ I don’t know	5
Number of family members	Less than 5	0
	More than 5	1

Note: Currency is SAR.

**Table 4:** Descriptive statistics for the sample.

Variable	N	Mode	SD
**Age**	182	3	1.20
**Gender**	182	1	0.48
**Level of Education**	182	4	1.41
**Monthly Income**	182	2	1.42
**Number of Family Members**	182	1	0.49

**Table 5:** Descriptive statistics for the survey items.

Code	Item	N	Mode	Mean	SD
**PC1**	In times of crisis, we can turn to each other for support.	182	3	3.28	1.00
**PC2**	We cry openly	182	3	2.71	0.99
**PC3**	We make sure members meet their family responsibilities.	182	3	2.76	0.95
**PC4**	We confide in each other.	182	3	2.81	0.99
**PC5**	Individuals are accepted for what they are.	182	3	2.79	0.97
**PC6**	People come right out and say things instead of hinting at them.	182	3	2.77	0.95
**PC7**	We resolve most everyday problems around the house.	182	3	2.79	0.96
**PC8**	When someone is upset the other one knows why.	182	3	2.80	0.95
**NC1**	Planning family activities is difficult because we misunderstand each other.	182	4	2.81	0.93
**NC2**	When you ask someone to do something, you have to check that they did it.	182	3	2.77	1.05
**NC3**	We are reluctant to show our affection for each other.	182	3	2.79	0.97
**NC4**	We cannot talk to each other about the sadness we feel.	182	3	2.79	0.96
**NC5**	You only get the interest of others when something is important to them.	182	4	2.73	1.01
**NC6**	You can’t tell how a person is feeling from what they are saying.	182	3	2.80	0.99
**NC7**	Some of us just don’t respond emotionally.	182	3	2.86	0.94
**NC8**	We avoid discussing our fears and concerns.	182	3	2.89	0.99

Note: 4 = Strongly agree, 3 = Agree, 2 = Disagree, 1 = Strongly disagree.

**Table 6:** Ordinal regression for positive communication.

	Coefficients	*SE*	*t* Stat.	*p*-Value	Lower 95%	Upper 95%
Intercept	2.458	0.267	9.209	0.000	1.931	2.984
Age	0.010	0.063	0.165	0.869	−0.144	0.135
Gender	−0.160	0.155	−1.029	0.305	−0.466	0.147
Education	0.080	0.053	1.516	0.131	−0.024	0.185
Income	0.063	0.053	1.191	0.235	−0.042	0.168
No. Family Members	−0.030	0.150	−0.198	0.844	−0.327	0.267

Note: See text for variable definitions.

**Table 7:** Ordinal regression for negative communication.

	Coefficients	Standard Error	*t* Stat.	*p*-Value	Lower 95%	Upper 95%
Intercept	2.087	0.267	10.947	0.000	2.279	3.335
Age	0.027	0.063	0.427	0.670	−0.098	0.152
Gender	0.003	0.156	0.017	0.986	−0.305	0.310
Education	−0.053	0.053	−0.991	0.323	−0.052	0.052
Income	0.039	0.053	0.726	0.469	−0.066	0.143
No. Family Members	0.105	0.151	0.698	0.486	−0.192	0.403

Note: See text for variable definitions.

## Data Availability

Due to the nature of this research, participants did not agree for their identified data to be shared publicly.
